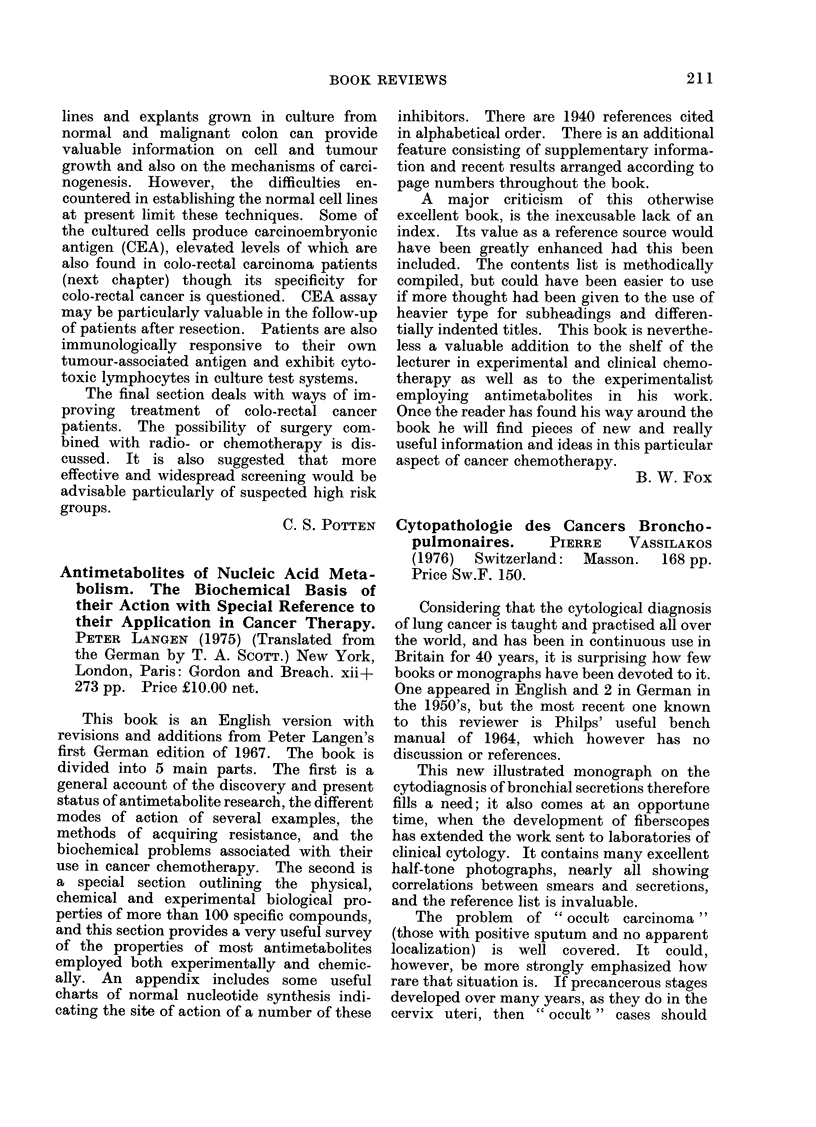# Antimetabolites of Nucleic Acid Metabolism. The Biochemical Basis of their Action with Special Reference to their Application in Cancer Therapy

**Published:** 1976-08

**Authors:** B. W. Fox


**Antimetabolites of Nucleic Acid Metabolism. The Biochemical Basis of their Action with Special Reference to their Application in Cancer Therapy.** Peter Langen (1975) (Translated from the German by T. A. Scott.) New York, London, Paris: Gordon and Breach. xii+273 pp. Price £10.00 net.

This book is an English version with revisions and additions from Peter Langen's first German edition of 1967. The book is divided into 5 main parts. The first is a general account of the discovery and present status of antimetabolite research, the different modes of action of several examples, the methods of acquiring resistance, and the biochemical problems associated with their use in cancer chemotherapy. The second is a special section outlining the physical, chemical and experimental biological properties of more than 100 specific compounds, and this section provides a very useful survey of the properties of most antimetabolites employed both experimentally and chemically. An appendix includes some useful charts of normal nucleotide synthesis indicating the site of action of a number of these inhibitors. There are 1940 references cited in alphabetical order. There is an additional feature consisting of supplementary information and recent results arranged according to page numbers throughout the book.

A major criticism of this otherwise excellent book, is the inexcusable lack of an index. Its value as a reference source would have been greatly enhanced had this been included. The contents list is methodically compiled, but could have been easier to use if more thought had been given to the use of heavier type for subheadings and differentially indented titles. This book is nevertheless a valuable addition to the shelf of the lecturer in experimental and clinical chemotherapy as well as to the experimentalist employing antimetabolites in his work. Once the reader has found his way around the book he will find pieces of new and really useful information and ideas in this particular aspect of cancer chemotherapy.

B. W. Fox